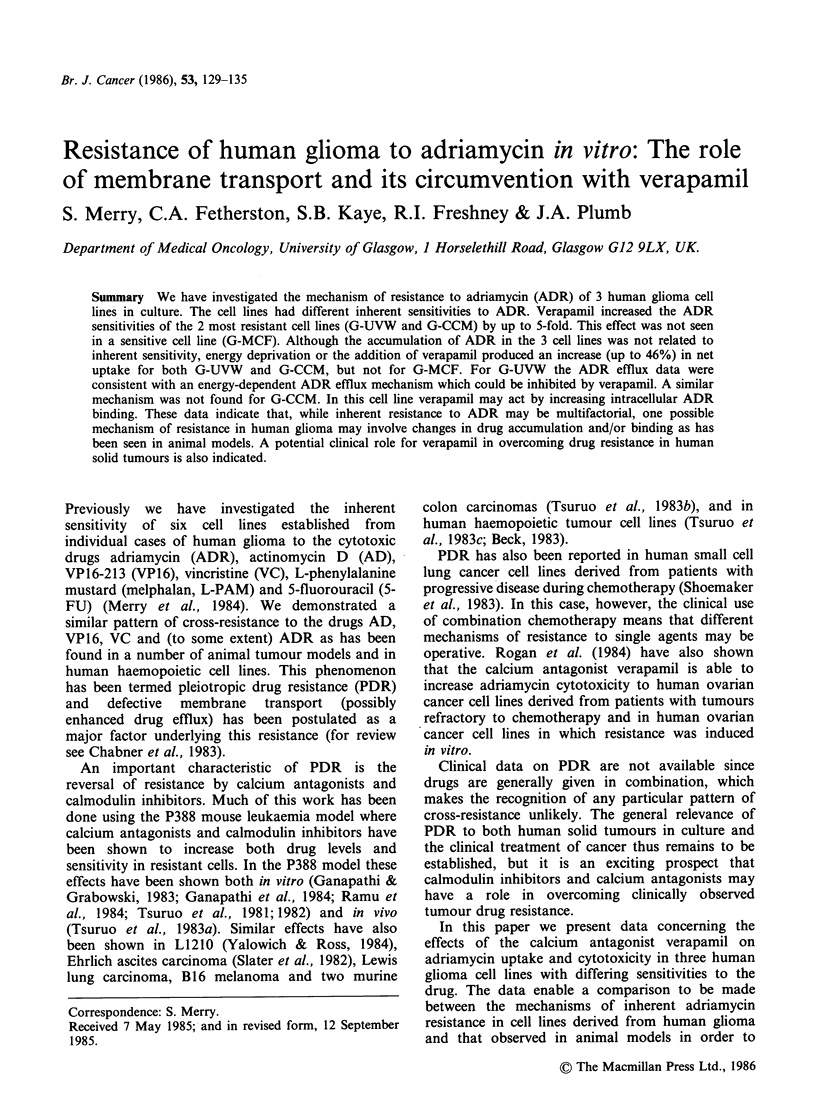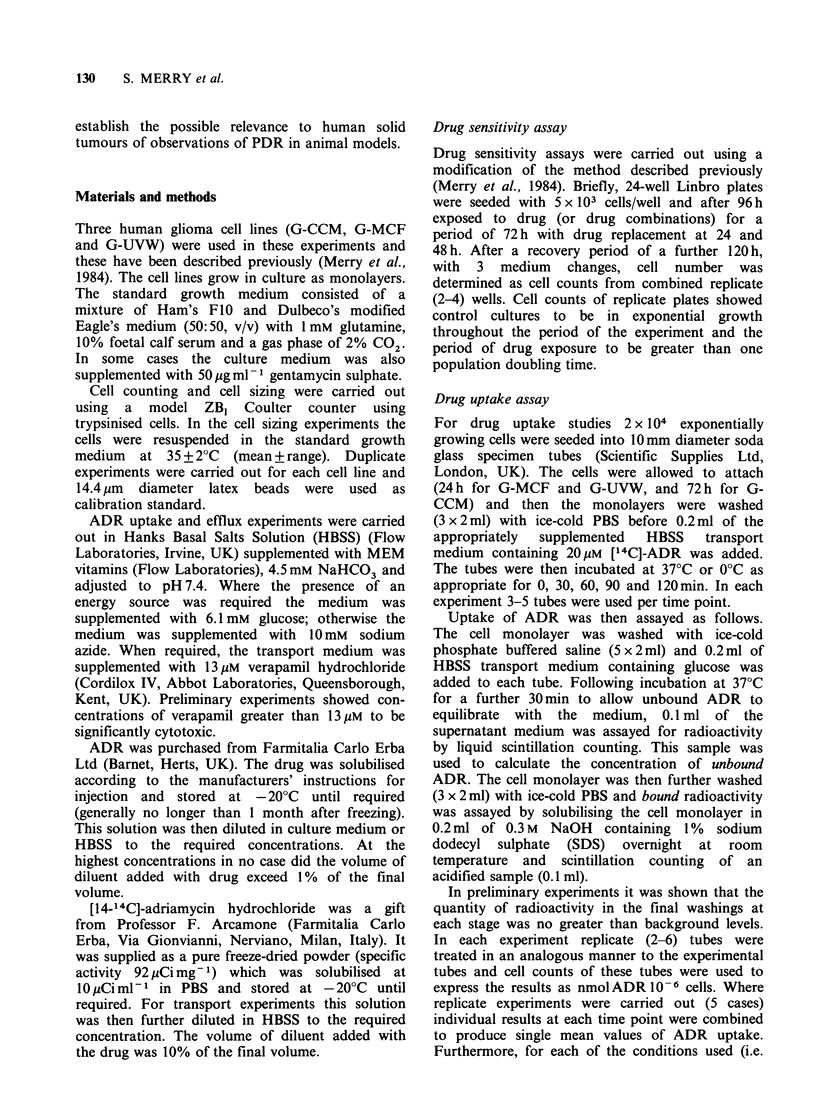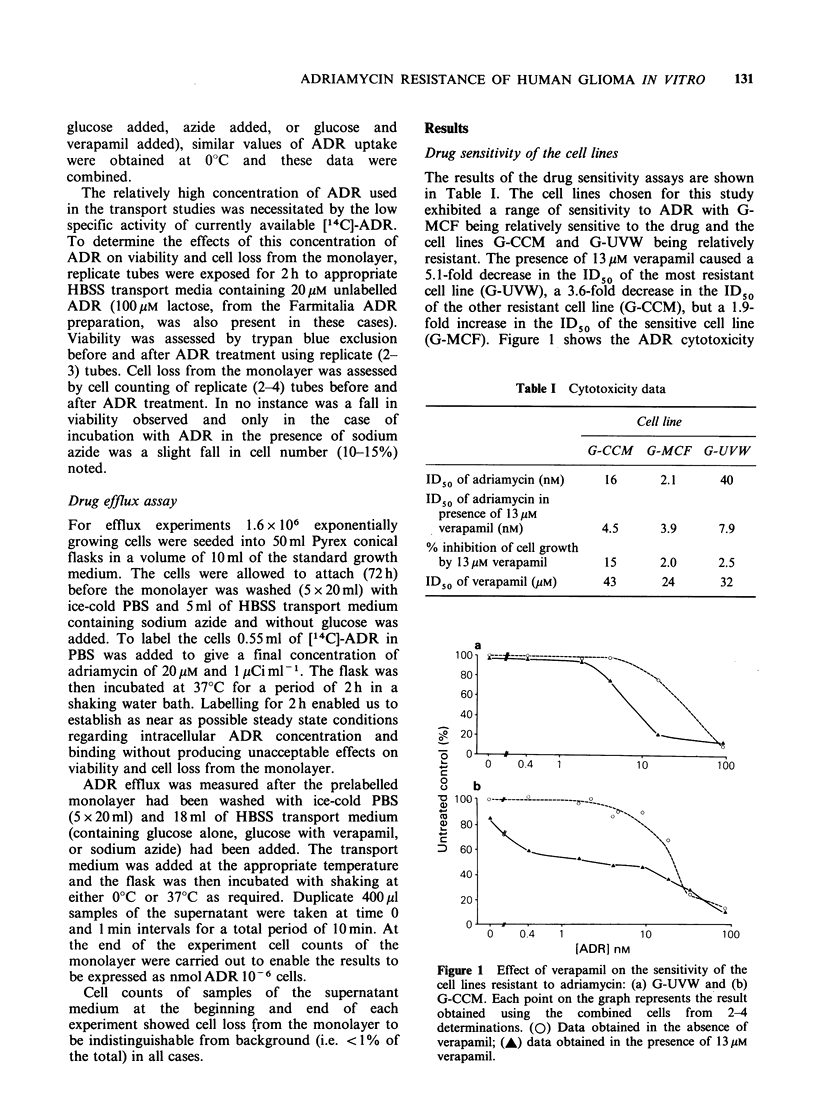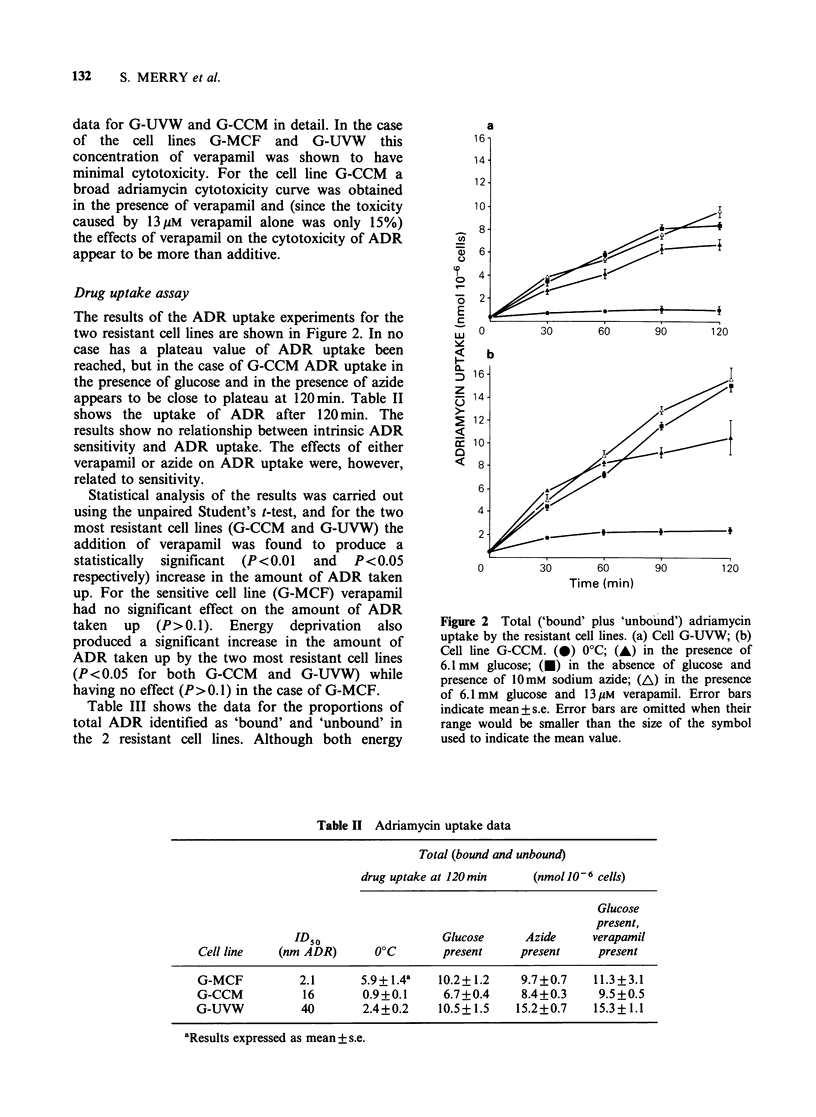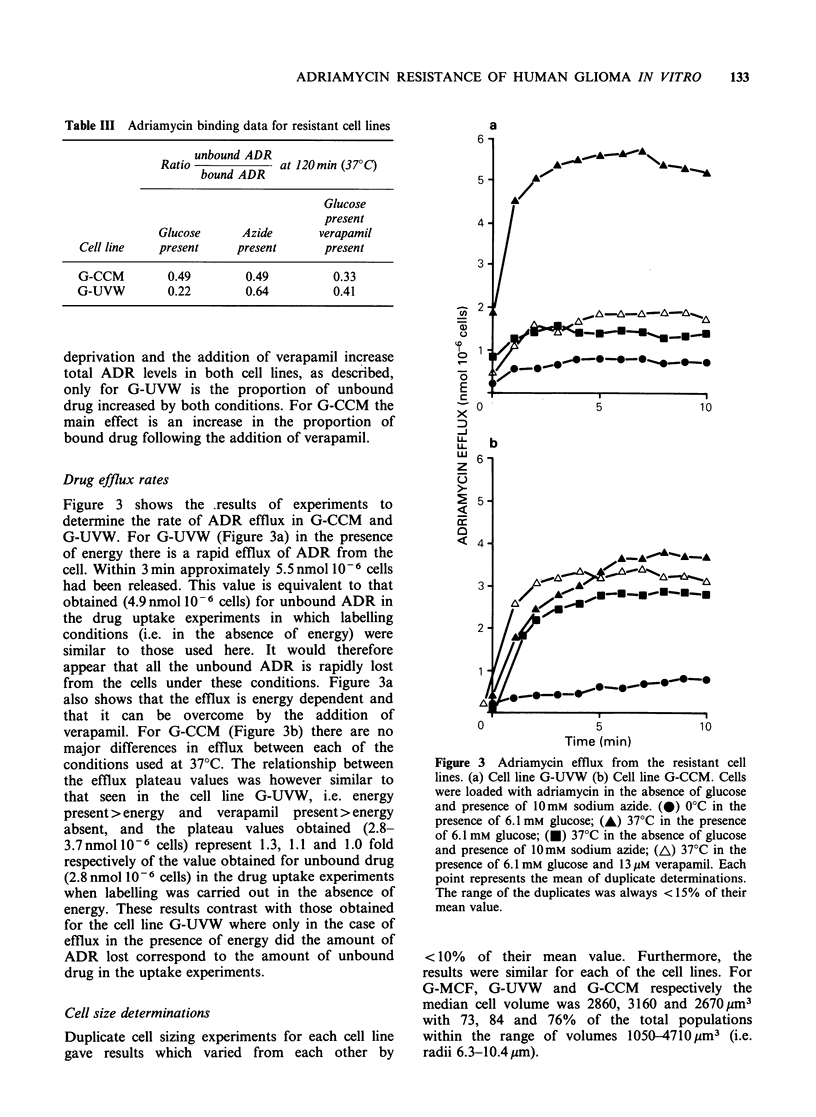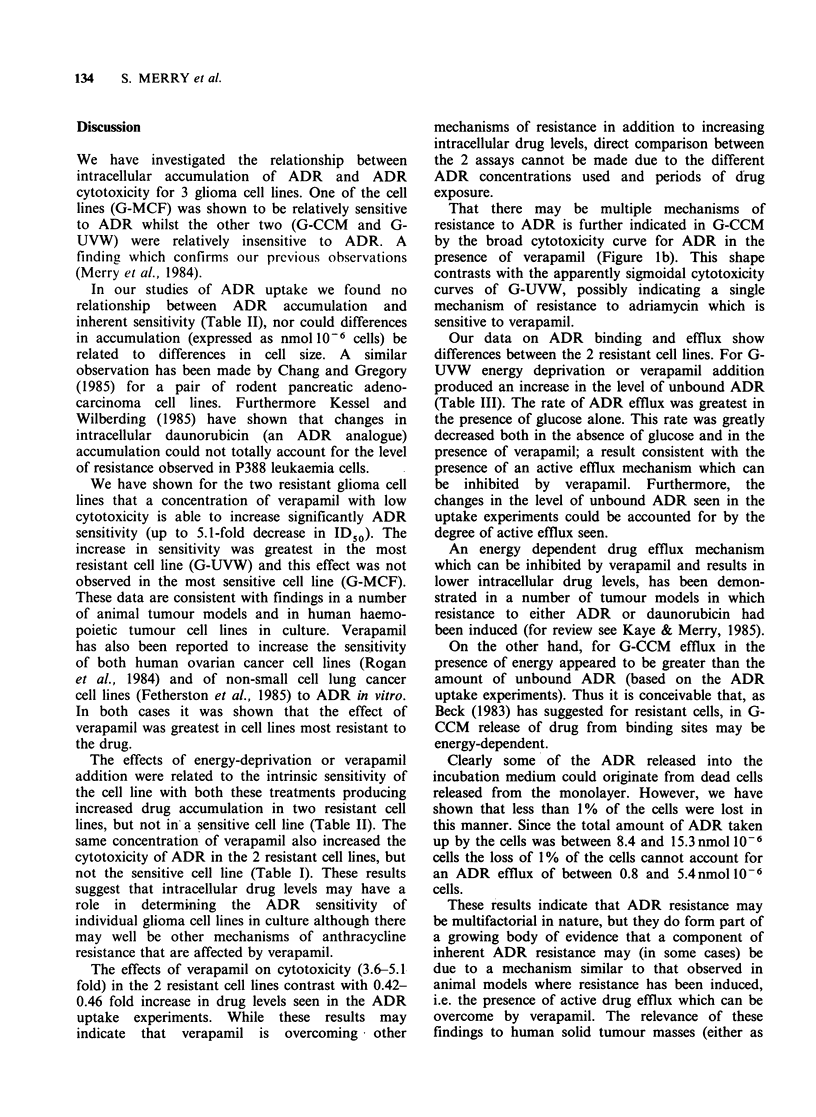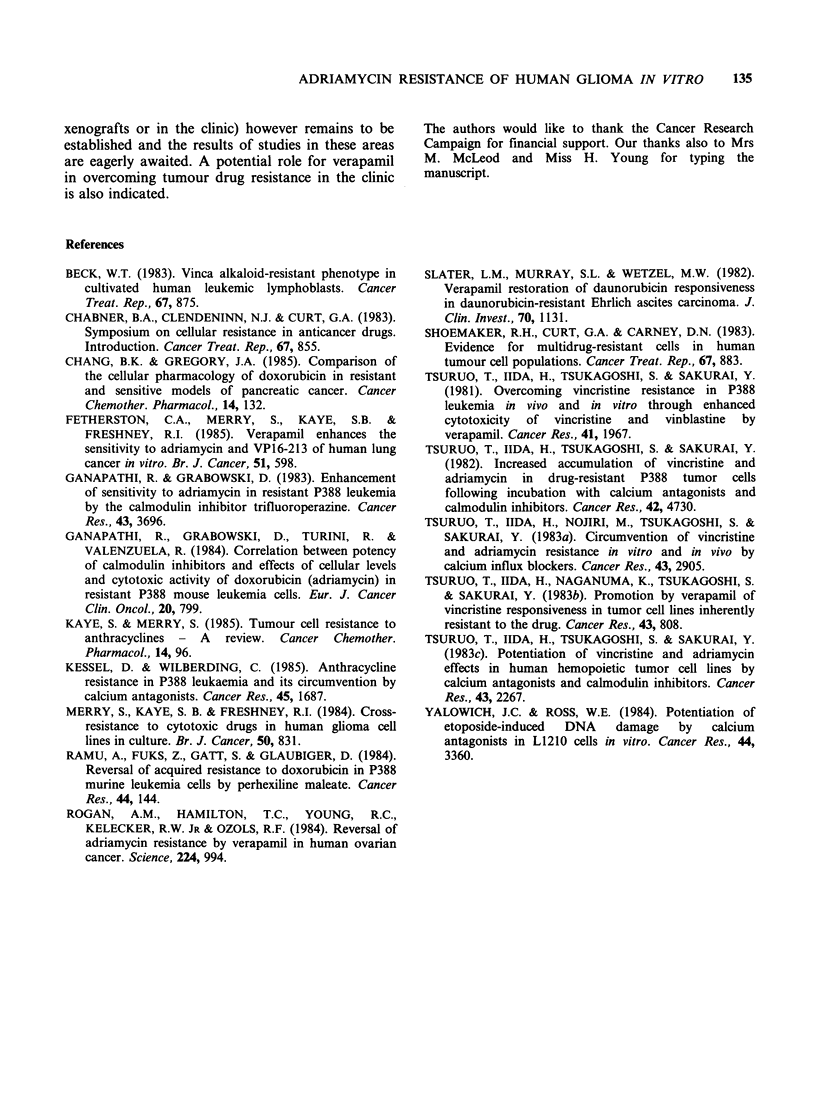# Resistance of human glioma to adriamycin in vitro: the role of membrane transport and its circumvention with verapamil.

**DOI:** 10.1038/bjc.1986.18

**Published:** 1986-01

**Authors:** S. Merry, C. A. Fetherston, S. B. Kaye, R. I. Freshney, J. A. Plumb

## Abstract

We have investigated the mechanism of resistance to adriamycin (ADR) of 3 human glioma cell lines in culture. The cell lines had different inherent sensitivities to ADR. Verapamil increased the ADR sensitivities of the 2 most resistant cell lines (G-UVW and G-CCM) by up to 5-fold. This effect was not seen in a sensitive cell line (G-MCF). Although the accumulation of ADR in the 3 cell lines was not related to inherent sensitivity, energy deprivation or the addition of verapamil produced an increase (up to 46%) in net uptake for both G-UVW and G-CCM, but not for G-MCF. For G-UVW the ADR efflux data were consistent with an energy-dependent ADR efflux mechanism which could be inhibited by verapamil. A similar mechanism was not found for G-CCM. In this cell line verapamil may act by increasing intracellular ADR binding. These data indicate that, while inherent resistance to ADR may be multifactorial, one possible mechanism of resistance in human glioma may involve changes in drug accumulation and/or binding as has been seen in animals models. A potential clinical role for verapamil in overcoming drug resistance in human solid tumours is also indicated.


Br. J. Cancer (1986), 53, 129–135

### Resistance of human glioma to adriamycin *in vitro*: The role of membrane transport and its circumvention with verapamil

S. Merry, C.A. Fetherston, S.B. Kaye, R.I. Freshney & J.A. Plumb

*Department of Medical Oncology, University of Glasgow, 1 Horselethill Road, Glasgow G12 9LX, UK.*

**Summary** We have investigated the mechanism of resistance to adriamycin (ADR) of 3 human glioma cell lines in culture. The cell lines had different inherent sensitivities to ADR. Verapamil increased the ADR sensitivities of the 2 most resistant cell lines (G-UVW and G-CCM) by up to 5-fold. This effect was not seen in a sensitive cell line (G-MCF). Although the accumulation of ADR in the 3 cell lines was not related to inherent sensitivity, energy deprivation or the addition of verapamil produced an increase (up to 46%) in net uptake for both G-UVW and G-CCM, but not for G-MCF. For G-UVW the ADR efflux data were consistent with an energy-dependent ADR efflux mechanism which could be inhibited by verapamil. A similar mechanism was not found for G-CCM. In this cell line verapamil may act by increasing intracellular ADR binding. These data indicate that, while inherent resistance to ADR may be multifactorial, one possible mechanism of resistance in human glioma may involve changes in drug accumulation and/or binding as has been seen in animal models. A potential clinical role for verapamil in overcoming drug resistance in human solid tumours is also indicated.

Previously we have investigated the inherent sensitivity of six cell lines established from individual cases of human glioma to the cytotoxic drugs adriamycin (ADR), actinomycin D (AD), VP16-213 (VP16), vincristine (VC), L-phenylalanine mustard (melphalan, L-PAM) and 5-fluorouracil (5-FU) (Merry *et al.*, 1984). We demonstrated a similar pattern of cross-resistance to the drugs AD, VP16, VC and (to some extent) ADR as has been found in a number of animal tumour models and in human haemopoietic cell lines. This phenomenon has been termed pleiotropic drug resistance (PDR) and defective membrane transport (possibly enhanced drug efflux) has been postulated as a major factor underlying this resistance (for review see Chabner *et al.*, 1983).

An important characteristic of PDR is the reversal of resistance by calcium antagonists and calmodulin inhibitors. Much of this work has been done using the P388 mouse leukaemia model where calcium antagonists and calmodulin inhibitors have been shown to increase both drug levels and sensitivity in resistant cells. In the P388 model these effects have been shown both *in vitro* (Ganapathi & Grabowski, 1983; Ganapathi *et al.*, 1984; Ramu *et al.*, 1984; Tsuruo *et al.*, 1981; 1982) and *in vivo* (Tsuruo *et al.*, 1983*a*). Similar effects have also been shown in L1210 (Yalowich & Ross, 1984), Ehrlich ascites carcinoma (Slater *et al.*, 1982), Lewis lung carcinoma, B16 melanoma and two murine colon carcinomas (Tsuruo *et al.*, 1983*b*), and in human haemopoietic tumour cell lines (Tsuruo *et al.*, 1983*c*; Beck, 1983).

PDR has also been reported in human small cell lung cancer cell lines derived from patients with progressive disease during chemotherapy (Shoemaker *et al.*, 1983). In this case, however, the clinical use of combination chemotherapy means that different mechanisms of resistance to single agents may be operative. Rogan *et al.* (1984) have also shown that the calcium antagonist verapamil is able to increase adriamycin cytotoxicity to human ovarian cancer cell lines derived from patients with tumours refractory to chemotherapy and in human ovarian cancer cell lines in which resistance was induced *in vitro*.

Clinical data on PDR are not available since drugs are generally given in combination, which makes the recognition of any particular pattern of cross-resistance unlikely. The general relevance of PDR to both human solid tumours in culture and the clinical treatment of cancer thus remains to be established, but it is an exciting prospect that calmodulin inhibitors and calcium antagonists may have a role in overcoming clinically observed tumour drug resistance.

In this paper we present data concerning the effects of the calcium antagonist verapamil on adriamycin uptake and cytotoxicity in three human glioma cell lines with differing sensitivities to the drug. The data enable a comparison to be made between the mechanisms of inherent adriamycin resistance in cell lines derived from human glioma and that observed in animal models in order to

Correspondence: S. Merry.
Received 7 May 1985; and in revised form, 12 September 1985.

© The Macmillan Press Ltd., 1986

establish the possible relevance to human solid tumours of observations of PDR in animal models.

### Materials and methods

Three human glioma cell lines (G-CCM, G-MCF and G-UVW) were used in these experiments and these have been described previously (Merry *et al.*, 1984). The cell lines grow in culture as monolayers. The standard growth medium consisted of a mixture of Ham's F10 and Dulbeco's modified Eagle's medium (50:50, v/v) with 1 mM glutamine, 10% foetal calf serum and a gas phase of 2% $CO_2$. In some cases the culture medium was also supplemented with $50\,\mu g\,ml^{-1}$ gentamycin sulphate.

Cell counting and cell sizing were carried out using a model $ZB_I$ Coulter counter using trypsinised cells. In the cell sizing experiments the cells were resuspended in the standard growth medium at $35 \pm 2°C$ (mean ± range). Duplicate experiments were carried out for each cell line and $14.4\,\mu m$ diameter latex beads were used as calibration standard.

ADR uptake and efflux experiments were carried out in Hanks Basal Salts Solution (HBSS) (Flow Laboratories, Irvine, UK) supplemented with MEM vitamins (Flow Laboratories), 4.5 mM $NaHCO_3$ and adjusted to pH 7.4. Where the presence of an energy source was required the medium was supplemented with 6.1 mM glucose; otherwise the medium was supplemented with 10 mM sodium azide. When required, the transport medium was supplemented with $13\,\mu M$ verapamil hydrochloride (Cordilox IV, Abbot Laboratories, Queensborough, Kent, UK). Preliminary experiments showed concentrations of verapamil greater than $13\,\mu M$ to be significantly cytotoxic.

ADR was purchased from Farmitalia Carlo Erba Ltd (Barnet, Herts, UK). The drug was solubilised according to the manufacturers' instructions for injection and stored at $-20°C$ until required (generally no longer than 1 month after freezing). This solution was then diluted in culture medium or HBSS to the required concentrations. At the highest concentrations in no case did the volume of diluent added with drug exceed 1% of the final volume.

[14-$^{14}$C]-adriamycin hydrochloride was a gift from Professor F. Arcamone (Farmitalia Carlo Erba, Via Gionvianni, Nerviano, Milan, Italy). It was supplied as a pure freeze-dried powder (specific activity $92\,\mu Ci\,mg^{-1}$) which was solubilised at $10\,\mu Ci\,ml^{-1}$ in PBS and stored at $-20°C$ until required. For transport experiments this solution was then further diluted in HBSS to the required concentration. The volume of diluent added with the drug was 10% of the final volume.

### *Drug sensitivity assay*

Drug sensitivity assays were carried out using a modification of the method described previously (Merry *et al.*, 1984). Briefly, 24-well Linbro plates were seeded with $5 \times 10^3$ cells/well and after 96 h exposed to drug (or drug combinations) for a period of 72 h with drug replacement at 24 and 48 h. After a recovery period of a further 120 h, with 3 medium changes, cell number was determined as cell counts from combined replicate (2–4) wells. Cell counts of replicate plates showed control cultures to be in exponential growth throughout the period of the experiment and the period of drug exposure to be greater than one population doubling time.

### *Drug uptake assay*

For drug uptake studies $2 \times 10^4$ exponentially growing cells were seeded into 10 mm diameter soda glass specimen tubes (Scientific Supplies Ltd, London, UK). The cells were allowed to attach (24 h for G-MCF and G-UVW, and 72 h for G-CCM) and then the monolayers were washed (3 x 2 ml) with ice-cold PBS before 0.2 ml of the appropriately supplemented HBSS transport medium containing $20\,\mu M$ [$^{14}$C]-ADR was added. The tubes were then incubated at 37°C or 0°C as appropriate for 0, 30, 60, 90 and 120 min. In each experiment 3–5 tubes were used per time point.

Uptake of ADR was then assayed as follows. The cell monolayer was washed with ice-cold phosphate buffered saline (5 x 2 ml) and 0.2 ml of HBSS transport medium containing glucose was added to each tube. Following incubation at 37°C for a further 30 min to allow unbound ADR to equilibrate with the medium, 0.1 ml of the supernatant medium was assayed for radioactivity by liquid scintillation counting. This sample was used to calculate the concentration of *unbound* ADR. The cell monolayer was then further washed (3 x 2 ml) with ice-cold PBS and *bound* radioactivity was assayed by solubilising the cell monolayer in 0.2 ml of 0.3 M NaOH containing 1% sodium dodecyl sulphate (SDS) overnight at room temperature and scintillation counting of an acidified sample (0.1 ml).

In preliminary experiments it was shown that the quantity of radioactivity in the final washings at each stage was no greater than background levels. In each experiment replicate (2–6) tubes were treated in an analogous manner to the experimental tubes and cell counts of these tubes were used to express the results as nmol ADR $10^{-6}$ cells. Where replicate experiments were carried out (5 cases) individual results at each time point were combined to produce single mean values of ADR uptake. Furthermore, for each of the conditions used (i.e.

glucose added, azide added, or glucose and verapamil added), similar values of ADR uptake were obtained at 0°C and these data were combined.

The relatively high concentration of ADR used in the transport studies was necessitated by the low specific activity of currently available [$^{14}$C]-ADR. To determine the effects of this concentration of ADR on viability and cell loss from the monolayer, replicate tubes were exposed for 2 h to appropriate HBSS transport media containing 20 $\mu$M unlabelled ADR (100 $\mu$M lactose, from the Farmitalia ADR preparation, was also present in these cases). Viability was assessed by trypan blue exclusion before and after ADR treatment using replicate (2–3) tubes. Cell loss from the monolayer was assessed by cell counting of replicate (2–4) tubes before and after ADR treatment. In no instance was a fall in viability observed and only in the case of incubation with ADR in the presence of sodium azide was a slight fall in cell number (10–15%) noted.

*Drug efflux assay*

For efflux experiments $1.6 \times 10^6$ exponentially growing cells were seeded into 50 ml Pyrex conical flasks in a volume of 10 ml of the standard growth medium. The cells were allowed to attach (72 h) before the monolayer was washed ($5 \times 20$ ml) with ice-cold PBS and 5 ml of HBSS transport medium containing sodium azide and without glucose was added. To label the cells 0.55 ml of [$^{14}$C]-ADR in PBS was added to give a final concentration of adriamycin of 20 $\mu$M and 1 $\mu$Ci ml$^{-1}$. The flask was then incubated at 37°C for a period of 2 h in a shaking water bath. Labelling for 2 h enabled us to establish as near as possible steady state conditions regarding intracellular ADR concentration and binding without producing unacceptable effects on viability and cell loss from the monolayer.

ADR efflux was measured after the prelabelled monolayer had been washed with ice-cold PBS ($5 \times 20$ ml) and 18 ml of HBSS transport medium (containing glucose alone, glucose with verapamil, or sodium azide) had been added. The transport medium was added at the appropriate temperature and the flask was then incubated with shaking at either 0°C or 37°C as required. Duplicate 400 $\mu$l samples of the supernatant were taken at time 0 and 1 min intervals for a total period of 10 min. At the end of the experiment cell counts of the monolayer were carried out to enable the results to be expressed as nmol ADR $10^{-6}$ cells.

Cell counts of samples of the supernatant medium at the beginning and end of each experiment showed cell loss from the monolayer to be indistinguishable from background (i.e. < 1% of the total) in all cases.

### Results

*Drug sensitivity of the cell lines*

The results of the drug sensitivity assays are shown in Table I. The cell lines chosen for this study exhibited a range of sensitivity to ADR with G-MCF being relatively sensitive to the drug and the cell lines G-CCM and G-UVW being relatively resistant. The presence of 13 $\mu$M verapamil caused a 5.1-fold decrease in the $ID_{50}$ of the most resistant cell line (G-UVW), a 3.6-fold decrease in the $ID_{50}$ of the other resistant cell line (G-CCM), but a 1.9-fold increase in the $ID_{50}$ of the sensitive cell line (G-MCF). Figure 1 shows the ADR cytotoxicity

**Table I** Cytotoxicity data

| | *Cell line* | | |
|---|---|---|---|
| | *G-CCM* | *G-MCF* | *G-UVW* |
| $ID_{50}$ of adriamycin (nM) | 16 | 2.1 | 40 |
| $ID_{50}$ of adriamycin in presence of 13 $\mu$M verapamil (nM) | 4.5 | 3.9 | 7.9 |
| % inhibition of cell growth by 13 $\mu$M verapamil | 15 | 2.0 | 2.5 |
| $ID_{50}$ of verapamil ($\mu$M) | 43 | 24 | 32 |

**Figure 1** Effect of verapamil on the sensitivity of the cell lines resistant to adriamycin: (a) G-UVW and (b) G-CCM. Each point on the graph represents the result obtained using the combined cells from 2–4 determinations. (○) Data obtained in the absence of verapamil; (▲) data obtained in the presence of 13 $\mu$M verapamil.

data for G-UVW and G-CCM in detail. In the case of the cell lines G-MCF and G-UVW this concentration of verapamil was shown to have minimal cytotoxicity. For the cell line G-CCM a broad adriamycin cytotoxicity curve was obtained in the presence of verapamil and (since the toxicity caused by 13 $\mu$M verapamil alone was only 15%) the effects of verapamil on the cytotoxicity of ADR appear to be more than additive.

### *Drug uptake assay*

The results of the ADR uptake experiments for the two resistant cell lines are shown in Figure 2. In no case has a plateau value of ADR uptake been reached, but in the case of G-CCM ADR uptake in the presence of glucose and in the presence of azide appears to be close to plateau at 120 min. Table II shows the uptake of ADR after 120 min. The results show no relationship between intrinsic ADR sensitivity and ADR uptake. The effects of either verapamil or azide on ADR uptake were, however, related to sensitivity.

Statistical analysis of the results was carried out using the unpaired Student's *t*-test, and for the two most resistant cell lines (G-CCM and G-UVW) the addition of verapamil was found to produce a statistically significant ($P < 0.01$ and $P < 0.05$ respectively) increase in the amount of ADR taken up. For the sensitive cell line (G-MCF) verapamil had no significant effect on the amount of ADR taken up ($P > 0.1$). Energy deprivation also produced a significant increase in the amount of ADR taken up by the two most resistant cell lines ($P < 0.05$ for both G-CCM and G-UVW) while having no effect ($P > 0.1$) in the case of G-MCF.

Table III shows the data for the proportions of total ADR identified as 'bound' and 'unbound' in the 2 resistant cell lines. Although both energy

**Figure 2** Total ('bound' plus 'unbound') adriamycin uptake by the resistant cell lines. (a) Cell G-UVW; (b) Cell line G-CCM. (●) 0°C; (▲) in the presence of 6.1 mM glucose; (■) in the absence of glucose and presence of 10 mM sodium azide; (△) in the presence of 6.1 mM glucose and 13 $\mu$M verapamil. Error bars indicate mean ± s.e. Error bars are omitted when their range would be smaller than the size of the symbol used to indicate the mean value.

**Table II** Adriamycin uptake data

| Cell line | $ID_{50}$ (nm ADR) | Total (bound and unbound) drug uptake at 120 min (nmol $10^{-6}$ cells): 0°C | Glucose present | Azide present | Glucose present, verapamil present |
|---|---|---|---|---|---|
| G-MCF | 2.1 | 5.9 ± 1.4[a] | 10.2 ± 1.2 | 9.7 ± 0.7 | 11.3 ± 3.1 |
| G-CCM | 16 | 0.9 ± 0.1 | 6.7 ± 0.4 | 8.4 ± 0.3 | 9.5 ± 0.5 |
| G-UVW | 40 | 2.4 ± 0.2 | 10.5 ± 1.5 | 15.2 ± 0.7 | 15.3 ± 1.1 |

[a]Results expressed as mean ± s.e.

**Table III** Adriamycin binding data for resistant cell lines

| | Ratio $\frac{unbound\ ADR}{bound\ ADR}$ at 120 min (37°C) | | |
|---|---|---|---|
| *Cell line* | *Glucose present* | *Azide present* | *Glucose present verapamil present* |
| G-CCM | 0.49 | 0.49 | 0.33 |
| G-UVW | 0.22 | 0.64 | 0.41 |

deprivation and the addition of verapamil increase total ADR levels in both cell lines, as described, only for G-UVW is the proportion of unbound drug increased by both conditions. For G-CCM the main effect is an increase in the proportion of bound drug following the addition of verapamil.

*Drug efflux rates*

Figure 3 shows the results of experiments to determine the rate of ADR efflux in G-CCM and G-UVW. For G-UVW (Figure 3a) in the presence of energy there is a rapid efflux of ADR from the cell. Within 3 min approximately 5.5 nmol $10^{-6}$ cells had been released. This value is equivalent to that obtained (4.9 nmol $10^{-6}$ cells) for unbound ADR in the drug uptake experiments in which labelling conditions (i.e. in the absence of energy) were similar to those used here. It would therefore appear that all the unbound ADR is rapidly lost from the cells under these conditions. Figure 3a also shows that the efflux is energy dependent and that it can be overcome by the addition of verapamil. For G-CCM (Figure 3b) there are no major differences in efflux between each of the conditions used at 37°C. The relationship between the efflux plateau values was however similar to that seen in the cell line G-UVW, i.e. energy present > energy and verapamil present > energy absent, and the plateau values obtained (2.8–3.7 nmol $10^{-6}$ cells) represent 1.3, 1.1 and 1.0 fold respectively of the value obtained for unbound drug (2.8 nmol $10^{-6}$ cells) in the drug uptake experiments when labelling was carried out in the absence of energy. These results contrast with those obtained for the cell line G-UVW where only in the case of efflux in the presence of energy did the amount of ADR lost correspond to the amount of unbound drug in the uptake experiments.

*Cell size determinations*

Duplicate cell sizing experiments for each cell line gave results which varied from each other by

**Figure 3** Adriamycin efflux from the resistant cell lines. (a) Cell line G-UVW (b) Cell line G-CCM. Cells were loaded with adriamycin in the absence of glucose and presence of 10 mM sodium azide. (●) 0°C in the presence of 6.1 mM glucose; (▲) 37°C in the presence of 6.1 mM glucose; (■) 37°C in the absence of glucose and presence of 10 mM sodium azide; (△) 37°C in the presence of 6.1 mM glucose and 13 $\mu$M verapamil. Each point represents the mean of duplicate determinations. The range of the duplicates was always < 15% of their mean value.

< 10% of their mean value. Furthermore, the results were similar for each of the cell lines. For G-MCF, G-UVW and G-CCM respectively the median cell volume was 2860, 3160 and 2670 $\mu m^3$ with 73, 84 and 76% of the total populations within the range of volumes 1050–4710 $\mu m^3$ (i.e. radii 6.3–10.4 $\mu$m).

### Discussion

We have investigated the relationship between intracellular accumulation of ADR and ADR cytotoxicity for 3 glioma cell lines. One of the cell lines (G-MCF) was shown to be relatively sensitive to ADR whilst the other two (G-CCM and G-UVW) were relatively insensitive to ADR. A finding which confirms our previous observations (Merry *et al.*, 1984).

In our studies of ADR uptake we found no relationship between ADR accumulation and inherent sensitivity (Table II), nor could differences in accumulation (expressed as nmol $10^{-6}$ cells) be related to differences in cell size. A similar observation has been made by Chang and Gregory (1985) for a pair of rodent pancreatic adenocarcinoma cell lines. Furthermore Kessel and Wilberding (1985) have shown that changes in intracellular daunorubicin (an ADR analogue) accumulation could not totally account for the level of resistance observed in P388 leukaemia cells.

We have shown for the two resistant glioma cell lines that a concentration of verapamil with low cytotoxicity is able to increase significantly ADR sensitivity (up to 5.1-fold decrease in $ID_{50}$). The increase in sensitivity was greatest in the most resistant cell line (G-UVW) and this effect was not observed in the most sensitive cell line (G-MCF). These data are consistent with findings in a number of animal tumour models and in human haemopoietic tumour cell lines in culture. Verapamil has also been reported to increase the sensitivity of both human ovarian cancer cell lines (Rogan *et al.*, 1984) and of non-small cell lung cancer cell lines (Fetherston *et al.*, 1985) to ADR *in vitro*. In both cases it was shown that the effect of verapamil was greatest in cell lines most resistant to the drug.

The effects of energy-deprivation or verapamil addition were related to the intrinsic sensitivity of the cell line with both these treatments producing increased drug accumulation in two resistant cell lines, but not in a sensitive cell line (Table II). The same concentration of verapamil also increased the cytotoxicity of ADR in the 2 resistant cell lines, but not the sensitive cell line (Table I). These results suggest that intracellular drug levels may have a role in determining the ADR sensitivity of individual glioma cell lines in culture although there may well be other mechanisms of anthracycline resistance that are affected by verapamil.

The effects of verapamil on cytotoxicity (3.6–5.1 fold) in the 2 resistant cell lines contrast with 0.42–0.46 fold increase in drug levels seen in the ADR uptake experiments. While these results may indicate that verapamil is overcoming other mechanisms of resistance in addition to increasing intracellular drug levels, direct comparison between the 2 assays cannot be made due to the different ADR concentrations used and periods of drug exposure.

That there may be multiple mechanisms of resistance to ADR is further indicated in G-CCM by the broad cytotoxicity curve for ADR in the presence of verapamil (Figure 1b). This shape contrasts with the apparently sigmoidal cytotoxicity curves of G-UVW, possibly indicating a single mechanism of resistance to adriamycin which is sensitive to verapamil.

Our data on ADR binding and efflux show differences between the 2 resistant cell lines. For G-UVW energy deprivation or verapamil addition produced an increase in the level of unbound ADR (Table III). The rate of ADR efflux was greatest in the presence of glucose alone. This rate was greatly decreased both in the absence of glucose and in the presence of verapamil; a result consistent with the presence of an active efflux mechanism which can be inhibited by verapamil. Furthermore, the changes in the level of unbound ADR seen in the uptake experiments could be accounted for by the degree of active efflux seen.

An energy dependent drug efflux mechanism which can be inhibited by verapamil and results in lower intracellular drug levels, has been demonstrated in a number of tumour models in which resistance to either ADR or daunorubicin had been induced (for review see Kaye & Merry, 1985).

On the other hand, for G-CCM efflux in the presence of energy appeared to be greater than the amount of unbound ADR (based on the ADR uptake experiments). Thus it is conceivable that, as Beck (1983) has suggested for resistant cells, in G-CCM release of drug from binding sites may be energy-dependent.

Clearly some of the ADR released into the incubation medium could originate from dead cells released from the monolayer. However, we have shown that less than 1% of the cells were lost in this manner. Since the total amount of ADR taken up by the cells was between 8.4 and 15.3 nmol $10^{-6}$ cells the loss of 1% of the cells cannot account for an ADR efflux of between 0.8 and 5.4 nmol $10^{-6}$ cells.

These results indicate that ADR resistance may be multifactorial in nature, but they do form part of a growing body of evidence that a component of inherent ADR resistance may (in some cases) be due to a mechanism similar to that observed in animal models where resistance has been induced, i.e. the presence of active drug efflux which can be overcome by verapamil. The relevance of these findings to human solid tumour masses (either as xenografts or in the clinic) however remains to be established and the results of studies in these areas are eagerly awaited. A potential role for verapamil in overcoming tumour drug resistance in the clinic is also indicated.

The authors would like to thank the Cancer Research Campaign for financial support. Our thanks also to Mrs M. McLeod and Miss H. Young for typing the manuscript.

### References

BECK, W.T. (1983). Vinca alkaloid-resistant phenotype in cultivated human leukemic lymphoblasts. *Cancer Treat. Rep.*, **67**, 875.

CHABNER, B.A., CLENDENINN, N.J. & CURT, G.A. (1983). Symposium on cellular resistance in anticancer drugs. Introduction. *Cancer Treat. Rep.*, **67**, 855.

CHANG, B.K. & GREGORY, J.A. (1985). Comparison of the cellular pharmacology of doxorubicin in resistant and sensitive models of pancreatic cancer. *Cancer Chemother. Pharmacol.*, **14**, 132.

FETHERSTON, C.A., MERRY, S., KAYE, S.B. & FRESHNEY, R.I. (1985). Verapamil enhances the sensitivity to adriamycin and VP16-213 of human lung cancer *in vitro*. *Br. J. Cancer*, **51**, 598.

GANAPATHI, R. & GRABOWSKI, D. (1983). Enhancement of sensitivity to adriamycin in resistant P388 leukemia by the calmodulin inhibitor trifluoroperazine. *Cancer Res.*, **43**, 3696.

GANAPATHI, R., GRABOWSKI, D., TURINI, R. & VALENZUELA, R. (1984). Correlation between potency of calmodulin inhibitors and effects of cellular levels and cytotoxic activity of doxorubicin (adriamycin) in resistant P388 mouse leukemia cells. *Eur. J. Cancer Clin. Oncol.*, **20**, 799.

KAYE, S. & MERRY, S. (1985). Tumour cell resistance to anthracyclines – A review. *Cancer Chemother. Pharmacol.*, **14**, 96.

KESSEL, D. & WILBERDING, C. (1985). Anthracycline resistance in P388 leukaemia and its circumvention by calcium antagonists. *Cancer Res.*, **45**, 1687.

MERRY, S., KAYE, S. B. & FRESHNEY, R.I. (1984). Cross-resistance to cytotoxic drugs in human glioma cell lines in culture. *Br. J. Cancer*, **50**, 831.

RAMU, A., FUKS, Z., GATT, S. & GLAUBIGER, D. (1984). Reversal of acquired resistance to doxorubicin in P388 murine leukemia cells by perhexiline maleate. *Cancer Res.*, **44**, 144.

ROGAN, A.M., HAMILTON, T.C., YOUNG, R.C., KELECKER, R.W. JR & OZOLS, R.F. (1984). Reversal of adriamycin resistance by verapamil in human ovarian cancer. *Science*, **224**, 994.

SLATER, L.M., MURRAY, S.L. & WETZEL, M.W. (1982). Verapamil restoration of daunorubicin responsiveness in daunorubicin-resistant Ehrlich ascites carcinoma. *J. Clin. Invest.*, **70**, 1131.

SHOEMAKER, R.H., CURT, G.A. & CARNEY, D.N. (1983). Evidence for multidrug-resistant cells in human tumour cell populations. *Cancer Treat. Rep.*, **67**, 883.

TSURUO, T., IIDA, H., TSUKAGOSHI, S. & SAKURAI, Y. (1981). Overcoming vincristine resistance in P388 leukemia *in vivo* and *in vitro* through enhanced cytotoxicity of vincristine and vinblastine by verapamil. *Cancer Res.*, **41**, 1967.

TSURUO, T., IIDA, H., TSUKAGOSHI, S. & SAKURAI, Y. (1982). Increased accumulation of vincristine and adriamycin in drug-resistant P388 tumor cells following incubation with calcium antagonists and calmodulin inhibitors. *Cancer Res.*, **42**, 4730.

TSURUO, T., IIDA, H., NOJIRI, M., TSUKAGOSHI, S. & SAKURAI, Y. (1983*a*). Circumvention of vincristine and adriamycin resistance *in vitro* and *in vivo* by calcium influx blockers. *Cancer Res.*, **43**, 2905.

TSURUO, T., IIDA, H., NAGANUMA, K., TSUKAGOSHI, S. & SAKURAI, Y. (1983*b*). Promotion by verapamil of vincristine responsiveness in tumor cell lines inherently resistant to the drug. *Cancer Res.*, **43**, 808.

TSURUO, T., IIDA, H., TSUKAGOSHI, S. & SAKURAI, Y. (1983*c*). Potentiation of vincristine and adriamycin effects in human hemopoietic tumor cell lines by calcium antagonists and calmodulin inhibitors. *Cancer Res.*, **43**, 2267.

YALOWICH, J.C. & ROSS, W.E. (1984). Potentiation of etoposide-induced DNA damage by calcium antagonists in L1210 cells *in vitro*. *Cancer Res.*, **44**, 3360.